# Malignancy risk in Australian rheumatoid arthritis patients treated with anti-tumour necrosis factor therapy: an update from the Australian Rheumatology Association Database (ARAD) prospective cohort study

**DOI:** 10.1186/s41927-018-0050-7

**Published:** 2019-01-08

**Authors:** Margaret P. Staples, Lyn March, Catherine Hill, Marissa Lassere, Rachelle Buchbinder

**Affiliations:** 1 Monash Department of Clinical Epidemiology, Cabrini Institute, Melbourne, Australia; 2 Department of Epidemiology and Preventive Medicine, School of Public Health and Preventive Medicine, Monash University, Melbourne, Australia; 3 Florance and Cope Professorial Department of Rheumatology, Royal North Shore Hospital, Institute of Bone and Joint Research, University of Sydney, Sydney, Australia; 4 The Queen Elizabeth and Royal Adelaide Hospitals, Adelaide, Australia; 5 Discipline of Medicine, University of Adelaide, Adelaide, Australia; 6 St George Hospital, University of New South Wales, Sydney, Australia

**Keywords:** Rheumatoid arthritis, Malignancy, Tumour necrosis factor, Biologic therapy

## Abstract

**Background:**

Tumour necrosis factor inhibitor (TNFi) therapy has been available for rheumatoid arthritis (RA) patients for several decades but data on the long-term risk of malignancy associated with its use is limited. Our aims were to assess malignancy risk in a cohort of Australian RA patients relative to the Australian population and to compare cancer risk for patients exposed to TNFi therapy versus a biologic-naïve group.

**Methods:**

Demographic data for RA participants enrolled in the Australian Rheumatology Association Database (ARAD) before 31 Dec 2012 were matched to national cancer records in May 2016 (linkage complete to 2012). Standardised incidence ratios (SIRs) were used to compare malignancy incidence in TNFi-exposed and biologic-naïve ARAD participants with the Australian general population using site-, age- and sex-specific rates by calendar year. Malignancy incidence in TNFi-exposed participants and biologic-naïve RA patients, were compared using rate ratios (RRs), adjusted for age, sex, smoking, methotrexate use and prior malignancy.

**Results:**

There were 107 malignancies reported after 10,120 person-years in the TNFi-exposed group (*N* = 2451) and 49 malignancies after 2232 person-years in the biologic-naïve group (*N* = 574). Compared with the general population, biologic-naïve RA patients showed an increased risk for overall malignancy (SIR 1.52 (95% confidence interval (CI) 1.16, 2.02) prostate cancer (SIR 2.10, 95% CI 1.18, 4.12). The risk of lung cancer was increased for both biologic naïve and TNFi-exposed patients compared with the general population (SIR 2.69 (95% CI 1.43 to 5.68) and SIR 1.69 (95% CI 1.05 to 2.90) respectively). For the TNFi-exposed patients there was an increased risk of lymphoid cancers (SIR 1.82, 95% CI 1.12, 3.18). There were no differences between the exposure groups in the risk of cancer for any of the specific sites examined.

**Conclusions:**

Overall malignancy incidence was elevated for biologic-naïve RA patients but not for those exposed to TNFi. TNFi exposure did not increase malignancy risk beyond that experienced by biologic-naïve patients. Lung cancer risk was increased for both TNFi-treated and biologic-naïve RA patients compared with the general population suggesting that RA status or RA treatments other than TNFi may be responsible in some way.

## Background

The true risk of malignancy in people with rheumatoid arthritis related to tumour necrosis factor inhibitor (TNFi) therapy remains uncertain, as TNF alpha can both promote cancer through inflammation as well as facilitate tumour cell death [1]. Systematic reviews of observational studies have generally not found an overall increased risk [2, 3], and this is true of most [4–9], but not all [10], systematic reviews based upon trial data.

As we have outlined previously [11], as well as the differing roles of TNF alpha depending upon the biologic circumstances, different biases may influence the estimates of malignancy risk depending upon the control group. For example comparison with the general population measures not only the effect of TNFi therapy but also the effect of having rheumatoid arthritis, which is associated with varying increased (e.g., lymphoma, lung cancer and renal cancer) or decreased (e.g., colorectal and breast cancer) risk of malignancy in its own right. Using TNFi naïve patients with rheumatoid arthritis as the control group partially controls for this but introduces other biases due to the fact that these patients may have less severe disease. While using pre-TNFi person years can partially control for disease severity, those with a history of malignancy are more likely to forgo TNFi therapy and this may result in an inflated estimated malignancy risk.

We previously reported an increased risk for melanoma and lung cancer in both biologic naïve and TNFi exposed RA patients compared with the Australian population but no increased risk associated with TNFi exposure for any of the sites examined [11]. There was also a possible reduction in risk of breast cancer for those exposed to TNFi compared with unexposed patients. However the malignancy incidence was low in our cohort suggesting that our study possibly lacked power. Larger numbers of patients followed for a longer time period may therefore be necessary to be completely confident that a true increase in malignancy risk in our setting does not exist.

The aim of this longer-term data linkage study was to therefore update our previous analysis of the risk of malignancy with TNFi therapy in a cohort of Australian rheumatoid arthritis patients compared with both the general population and a biologic-naïve group of rheumatoid arthritis patients.

## Methods

### Setting

As outlined previously [11], government-subsidised treatment with biological disease modifying anti-rheumatic drugs is subject to strict eligibility criteria in Australia. While these criteria have changed over time, they are designed to limit bDMARD therapy to patients with highly active and treatment-resistant RA. To be eligible, patients must have demonstrated an inadequate response over a period of 6 months to at least two traditional DMARDs including methotrexate (unless contraindicated), a tender and swollen joint count of greater than 20 joints (or 4 large joints) and elevated inflammatory markers. Unless absolute contra-indications were present, TNFi therapy was the mandatory first choice biologic therapy until November 2007.

### Australian rheumatology association database (ARAD) design and data collection

The ARAD design, content and governance has been described previously [11–15]. In brief, It is a voluntary national registry, established in 2001, that collects longitudinal health outcomes data from Australian patients with inflammatory arthritis. Enrolment can occur at any time and includes patients who have not ever been exposed to biologic therapy, but most often occurs at the time a patient commences biologics. 246 (79%) rheumatologists from all states and territories having contributed patients and it appears nationally representative based on participant residential postcode, demographic and clinical characteristics [15].

Twenty local ethics committees and organisations across all Australian states and territories have granted ethical approval. All participants provide written permission to be contacted by ARAD investigators and written informed consent to participate in the registry and the associated linkages.

At enrolment, the treating rheumatologist provides details of diagnosis, disease status data (ESR, CRP and joint count) and the biologic prescribed (if applicable). Detailed questionnaires which are either paper-based or online, are completed by participants at enrolment and then at six- to 12-monthly intervals thereafter. Participant data collected include demographic details, smoking and alcohol history, disease duration and severity, self-reported past and current medical history including other chronic conditions, use of anti-rheumatic drugs and their commencement date, Assessment of Quality of Life (AQoL) [16] and arthritis-specific disability assessed by the Health Assessment Questionnaire (HAQ) [17], the EQ5D [18] and the SF 36 [19].

### Eligibility for this study

To be eligible for this updated analysis, inclusion criteria were rheumatologist-diagnosed rheumatoid arthritis and enrolment in ARAD prior to 31 December 2012 (the analysis cut-off date). As before, participants were divided into two mutually exclusive groups comprising biologic-naïve participants for the entire duration of observation, and participants who commenced a TNFi (etanercept, adalimumab, infliximab, golimumab or certolizumab) as their first line biologic therapy. This contrasts with our original analysis which only included those who had commenced etanercept, adalimumab and infliximab as subsidised prescription of certolizumab and golimumab was not available at that time. We excluded participants who commenced a non-TNFi biologic as first line therapy.

For included participants, baseline demographic and clinical, disability and quality of life data were extracted from the questionnaire completed at ARAD enrolment (baseline questionnaire). Some questions relating to baseline clinical variables were incomplete as these question were only added in January 2006.

### Ascertainment of malignancy

As outlined previously [11], notification for all invasive cancers except non-melanoma skin cancers to state-based cancer registries has been mandatory in Australia since 1982 and these data are aggregated to national level by the National Cancer Statistics Clearing House (NCSCH) [20]. Site of malignancy is coded by The International Classification of Diseases, 10th Revision (ICD-10). Linkage of the ARAD patient database with the NCSCH took place in May 2016, and at that time reporting of malignancies was complete for 1982 to 2012. We were therefore able to ascertain all malignancies that occurred in ARAD participants from the time of their enrolment in ARAD. The date of diagnosis of malignancy as recorded in the NCSCH was used in the analysis.

### Data analysis

Person-years for biologic-naïve patients were calculated from the date of enrolment in ARAD until death or analysis cut-off date (31 December 2012). Person years of exposure to a TNFi began at either the start date of the TNFi therapy or enrolment in ARAD (whichever was later) and continued until death or analysis cut-off date.

As before [11], standardised incidence ratios (SIRs) were used to compare the incidence of malignancy in biologic naïve and the TNFi-exposed ARAD participants with incidence in the Australian general population using the site, age, and sex and calendar year specific incidence rates as published by the Australian Institute of Health and Welfare [21]. Malignancy risk in TNFi-exposed participants was compared with that in biologic naive participants using rate ratios calculated using the Mantel-Cox method with significance assessed using the log-rank test for the respective ‘time to malignancy’ distributions adjusted for age, sex, calendar year, smoking status, prior malignancy and methotrexate use. All data were analysed using Stata 14 [22].

## Results

There were 3025 RA patients eligible for study inclusion; 574 were biologic-naïve and 2451 had received TNFi therapy (first-line therapy: etanercept (*n* = 1302, 53%), adalimumab (*n* = 900, 37%), infliximab (*n* = 152, 6%), golimumab (*n* = 67, 3%), certolizumab (*n* = 30, 1%). The characteristics of the biologic naïve group at entry to ARAD and the TNFi-exposed group at commencement of therapy are shown in Table 1.

**Table 1:** Baseline characteristics of biologic-naïve and TNFi-exposed participants

	Biologic naïve (*N* = 574)			TNFi-exposed (*N* = 2451)			*p*-value
	n		%	n		%	
Female, n (%)	402		70	1810		73.9	0.06
Rheumatoid factor positive (*n* = 444:1925)	386		84.9	1625		82	0.012
Cyclic citrullinated peptide (CCP) + ve (*n* = 13:109)	10		76.9	74		67.9	0.51
No. of prior DMARDs							< 0.001
None	31		5.4	200		8.2	
1	95		16.6	53		2.2	
2	154		26.5	132		5.4	
3	115		20	408		16.7	
4	92		16	628		25.6	
5	59		10.3	583		23.8	
6–10	28		4.9	447		18.2	
Ever used methotrexate	493		85.9	2185		89.2	0.03
Ever used prednisolone	318		55.4	1933		78.9	< 0.001
Current smoker	78		13.6	341		14.1	0.76
Prior malignancy	44		7.4	94		3.7	< 0.001
	N	Mean	SD	N	Mean	SD	p-value
Age (years)	574	62.4	12.3	2451	55.7	12.4	< 0.001
HAQ (Range 0–3)	572	1.1	0.7	2443	1.2	0.7	< 0.001
AQoL (Range − 0.04 – 1)	572	0.6	0.3	2438	0.5	0.2	< 0.001
EQ5D (UK) (Range − 0.59 - 1)	560	0.7	0.3	2405	0.6	0.3	< 0.001
PCS (Range 0–100)	556	35.1	11.2	2374	32.1	11	< 0.001
MCS (Range 0–100)	556	48.3	11.6	2374	46.5	11.9	0.001
ESR (Range 2–109)	150	26.3	22.1	1259	33.2	25.1	0.002
CRP (Range 0.4–128)	155	17.1	22.6	1260	25.3	30.1	0.001
Joint count (Range 0–50)	150	12.2	12.8	1323	23.5	11	0.001
Years since RA diagnosis	566	13.8	11.6	2444	14.1	10.6	0.1

Those starting TNFi as a first biologic therapy were younger (55.7 vs 62.4 years) and had more active disease, greater disability and poorer quality of life compared with the biologic naïve participants. They also had greater prior use of DMARDs. The median time since diagnosis with RA was similar for both groups, while slightly more biologic naïve participants were rheumatoid factor positive (84.9% versus 82%). Prior malignancies were more common among the biologic naïve group (7.4% compared with 3.7% in the TNFi exposed group).

Follow up was 10,120 and 2232 person-years for TNFi-exposed and biologic-naïve patients respectively. There were 107 malignancies recorded in the TNFi-exposed group and 49 in the biologic-naïve group. The overall malignancy risk for biologic-naïve participants was higher than that expected on the basis of population rates (SIR 1.5, 95% CI 1.2, 2.0). On examination of site specific cancers, these patients had an elevated risk of lung (SIR 2.7, 95% CI (confidence interval) 1.4, 5.7) and prostate (SIR 2.1, 95% CI 1.2, 4.1) cancers (Table 2).

**Table 2:** Cancer risk compared with the general population and relative risk in the TNFi-exposed vs biologic naïve participants

	Risk in biologic-naïve RA patients			Risk in TNFi-exposed patients			TNFi-exposed vs biologic-naïve patients
	Observed	Expected	SIR* (95% CI)	Observed	Expected	SIR (95% CI)	RR (95% CI)
All invasive cancers	49	32.27	1.52 (1.16, 2.02)	107	102.62	1.04 (0.87, 1.27)	0.71 (0.46, 1.08)
Melanoma	4	2.65	1.51 (0.57, 5.35)	12	9.17	1.31 (0.76, 2.46)	1.18 (0.29, 4.70)
Lung	9	3.35	2.69 (1.43, 5.68)	16	9.45	1.69 (1.05, 2.90)	0.38 (0.12, 1.20)
Lymphoid cancers	5	2.71	1.84 (0.78, 5.47)	15	8.24	1.82 (1.12, 3.18)	0.79 (0.25, 2.55
Bowel	7	4.59	1.53 (0.74, 3.66)	10	13.11	0.76 (0.42, 1.54)	0.75 (0.17, 3.29)
Prostate	10	4.75	2.10 (1.18, 4.12)	12	13.67	0.88 (0.51, 1.64)	0.67 (0.23, 1.99)
Breast	8	4.58	1.75 (0.90, 3.86)	17	19.35	0.88 (0.56, 1.47)	0.50 (0.22, 1.16)

*SIR* Standardised Incidence Ratio, *CI* confidence interval, *RR* relative risk

By contrast, the overall malignancy risk among TNFi-treated RA patients was not elevated in comparison with the general population (Table 2), but there was an increased risk for lung (SIR 1.7, 95% CI 1.1, 2.9) and lymphoid (SIR 1.8, 95% CI1.1, 3.2) cancers. The risk of melanoma was not increased for either exposure group relative to the general Australian population. When the TNFi-treated RA patients were compared with the biologic-naïve RA cohort, there were no significant differences in malignancy risk overall or for any of the specific cancer sites examined.

## Discussion

We found an overall increased malignancy risk for the biologic-naïve patients compared with the general population, but there was no evidence for an increased risk in the TNFi-treated RA patients compared with the general population. This result is consistent with numerous previously reported observational studies [2, 23–31].

The magnitude of the increased SIRs for lymphoid cancers was similar for both exposure groups but smaller numbers in the biologic-naïve group resulted in reduced precision and wide confidence intervals around this estimate. Both lymphomas and lung cancers have been shown to be elevated in RA patients, compared to the general population [32]. While the finding of an increased risk of lung cancer in biologic-naïve RA patients compared with the general population is consistent with previous studies including our own original report [11], we also identified an increased risk of lung cancer related to TNFi exposure, unlike other registry studies [33, 34], and our prior analysis [11]. As smoking rates in the ARAD cohort are comparable with the national smoking rates in Australia of 14.5% [35], the elevated risk for both exposure groups suggests that RA status and background treatments may be an explanation. However this is a new finding and needs to be verified with longer follow up of our cohort and other national registries.

Australia has high background rates of melanoma but we did not find an increased risk of melanoma in either the TNFi-naïve or exposed group relative to these higher rates in the current analysis. Our previous study [11], based on fewer events and shorter follow-up, reported an increased risk of melanoma in both exposure groups that was not evident in this analysis. The higher number of events in both groups and the longer follow-up will result in better precision and a reduction in possible bias in the estimates for this study.

There is conflicting evidence regarding the risk of melanoma in rheumatoid arthritis patients treated with TNFi therapy [29, 34, 36–39]. For example, Wolfe et al. reported an elevated risk of melanoma in rheumatoid arthritis patients overall but no increase in risk after exposure to biologic therapy [34], while Raaschou et al. found no increase in risk of melanoma in biologic-naïve individuals but an increase in risk for those treated with TNFi therapy [36]. A review and pooled analysis by Perkins et al. of 11 studies from Europe, North America and Asia Pacific comparing the risk of melanoma in biologic-naïve patients with the general population found no increased risk [37], while another more recent pooled analysis of data from 11 biologic registers from nine European countries found no evidence of an increased risk of melanoma in biologic-naïve or TNFi-exposed patients [38]. As we noted previously [11], data regarding exposure to specific medications such as methotrexate, and other participant characteristics, such as genetic background including skin colour and relative ultraviolet light exposure, may also be important for melanoma risk and if feasible should be considered in future analyses.

We did not confirm our previously reported reduced risk of colorectal cancer in the TNFi-exposed group. Our earlier estimate was based on a single case in the exposed group while this current study included 10 cases. The lower proportion of prior malignancies in the TNFi-exposed group is in keeping with earlier guidance for rheumatologists to avoid using TNFi therapy in patients with a previous cancer or those they perceive to be at higher risk of cancer or precancerous lesions such as rectal polyps or breast ductal carcinoma in situ. An artificially low incidence of malignancy in the exposed group could result in an underestimate of malignancy risk in the TNFi-exposed group. As the results of long term longitudinal data look more reassuring about malignancy risk and TNFi therapy is used by a greater proportion of people with rheumatoid arthritis, it will be important to continue monitoring these associations to be reassured that this is not an underestimate of real risk and confounded by indication.

Strengths of this updated analysis of malignancy risk based upon a national Australian registry include national representation of patients from most rheumatologists, over 12,000 person years of follow up, and virtual complete ascertainment of malignancies between 1982 and the end of 2012 due to mandatory reporting. It would be ideal to have national data for cancer risk among individuals with rheumatoid arthritis but in its absence we compared malignancy risk of TNFi-exposed individuals to both the general population and TNFi-naïve rheumatoid arthritis patients. As outlined in our introduction, these analyses are each prone to different forms of bias. While the comparison with biologic-naïve patients controls for the potential confounding of having the disease, the lower levels of disease activity in biologic-naïve patients could falsely inflate the risk of malignancy associated with TNFi therapy.

Participation in ARAD remains voluntary and while baseline characteristics of ARAD participants with rheumatoid arthritis are similar to cohorts in other national registries, it is not known whether or not our TNFi-treated cohort is representative of all Australian TNFi-treated patients. As also acknowledged in our previous paper [11], our analyses may also be compromised by immortal time bias as patients already diagnosed with cancer may not enrol in ARAD.

Our updated analysis may still lack power despite an increase in patient years of follow-up. We also had insufficient patient numbers to analyse data for each TNFi separately. While we were able to include data for the anti-TNF therapies certolizumab and golimumab in this analysis, we still have too few cases and limited follow-up for these patients to ensure generalisability of our findings to these drugs. As before [11], we cannot explore in detail the effect of methotrexate and other disease-modifying anti-rheumatic drugs on malignancy risk as we do not have sufficiently detailed information precise duration and doses of concomitant and prior medications.

## Conclusions

Our updated analysis adds to the body of evidence suggesting that TNFi exposure is not associated with an overall increased risk of malignancy in people with rheumatoid arthritis. As experience with these drugs increases and more data become available regarding risks associated with longer term exposure, a clearer picture has begun to emerge suggesting that TNFi therapy does not increase malignancy risk beyond that experienced by biologic naïve RA patients. We still consider it prudent to regularly monitor patients for development of all types of skin cancers, as while not assessed in this study, an increased risk of non-melanoma skin cancers has been reported across several studies (40). It can take many years for exposure to carcinogens to become apparent and further monitoring of rheumatoid arthritis patients on these drugs is recommended.

## Abbreviations

AQoL: Assessment of Quality of Life
ARAD: Australian Rheumatology Association Database
bDMARD: biologic disease modifying anti-rheumatic drug
CI: Confidence interval
CRP: C reactive protein
DMARD: disease modifying anti-rheumatic drug
ESR: erythrocyte sedimentation rate
HAQ: Health Assessment Questionnaire
NCSCH: National Cancer Statistics Clearing House
RA: Rheumatoid arthritis
RR: Relative risk
SF 36: 36-Item Short Form Health Survey
SIR: Standardised incidence ratio
TNFi: Tumour necrosis factor inhibitor

## References

[1] Lebrec H, Ponce R, Preston B, Iles J, Born T, Hooper M (2015). Tumor necrosis factor, tumor necrosis factor inhibition, and cancer risk. Curr Med Res Op.

[2] Mariette X, Matucci-Cerinic M, Pavelka K, Taylor P, van Vollenhoven R, Heatley R (2011). Malignancies associated with tumour necrosis factor inhibitors in registries and prospective observational studies: a systematic review and meta-analysis. Ann Rheum Dis.

[3] Ramiro S, Sepriano A, Chatzidionysiou K, Nam JL, Smolen JS, van der Heijde D (2017). Safety of synthetic and biological DMARDs: a systematic literature review informing the 2016 update of the EULAR recommendations for management of rheumatoid arthritis. Ann Rheum Dis.

[4] Alonso-Ruiz A, Pijoan JI, Ansuategui E, Urkaregi A, Calabozo M, Quintana A (2008). Tumor necrosis factor alpha drugs in rheumatoid arthritis: systematic review and metaanalysis of efficacy and safety. BMC Musculoskel Dis.

[5] Leombruno JP, Einarson TR, Keystone EC (2009). The safety of anti-tumour necrosis factor treatments in rheumatoid arthritis: meta and exposure-adjusted pooled analyses of serious adverse events. Ann Rheum Dis.

[6] Lopez-Olivo MA, Tayar JH, Martinez-Lopez JA, Pollono EN, Cueto JP, Gonzales-Crespo MR (2012). Risk of malignancies in patients with rheumatoid arthritis treated with biologic therapy: a meta-analysis. JAMA.

[7] Michaud TL, Rho YH, Shamliyan T, Kuntz KM, Choi HK (2014). The comparative safety of tumor necrosis factor inhibitors in rheumatoid arthritis: a meta-analysis update of 44 trials. Am J Med.

[8] Thompson AE, Rieder SW, Pope JE (2011). Tumor necrosis factor therapy and the risk of serious infection and malignancy in patients with early rheumatoid arthritis: a meta-analysis of randomized controlled trials. Arthritis Rheum.

[9] Askling J, Fahrbach K, Nordstrom B, Ross S, Schmid CH, Symmons D (2011). Cancer risk with tumor necrosis factor alpha (TNF) inhibitors: meta-analysis of randomized controlled trials of adalimumab, etanercept, and infliximab using patient level data. Pharmacoepidemiol Drug Saf.

[10] Bongartz T, Sutton AJ, Sweeting MJ, Buchan I, Matteson EL, Montori V (2006). Anti-TNF antibody therapy in rheumatoid arthritis and the risk of serious infections and malignancies: systematic review and meta-analysis of rare harmful effects in randomized controlled trials. JAMA.

[11] Buchbinder R, van Doornum S, Staples M, Lassere M, March L (2015). Malignancy risk in Australian rheumatoid arhritis patients treated with anti-tumour necrosis factor therapy: analysis of the Australian rheumatology association database (ARAD) prospective cohort study. BMC Musculoskel Dis..

[12] Briggs A, March L, Lassere M, Reid C, Henderson L, Murphy B (2009). Baseline comorbidities in a population-based cohort of rheumatoid arthritis receiving biological therapy: data from the Australian rheumatology association database. Int J Rheumatol.

[13] Buchbinder R, March L, Lassere M, Briggs AM, Portek I, Reid C (2007). Effect of treatment with biological agents for arthritis in Australia: the Australian rheumatology association database. Intern Med J.

[14] Staples MP, March L, Lassere M, Reid C, Buchbinder R (2011). Health-related quality of life and continuation rate on first-line anti-tumour necrosis factor therapy among rheumatoid arthritis patients from the Australian rheumatology association database. Rheumatol.

[15] Williams MP, Buchbinder R, March L, Lassere M (2011). The Australian rheumatology association database (ARAD). Sem Arthritis Rheum..

[16] Hawthorne G, Richardson J, Osborne R (1999). The assessment of quality of life (AQoL) instrument: a psychometric measure of health related quality of life. Quality Life Res.

[17] Fries JF, Spitz PW, Young DY (1982). The dimensions of health outcomes: the health assessment questionnaire, disability and pain scales. J Rheumatol.

[18] Rabin R, de Charro F (2001). EQ-5D: a measure of health status from the EuroQol group. Ann Med.

[19] Ware JE, Sherbourne CD (1992). The MOS 36-item short-form health survey (SF-36). I Conceptual framework and item selection. Med Care.

[20] Australian Institute of Health and Welfare (2013). National Cancer Statistics Clearing House Protocol 2013.

[21] Australian Institute of Health and Welfare (2017). Australian Cancer incidence and mortality (ACIM) books.

[22] Stata Corporation (2006). Stata 10. Stata 14.2 ed.

[23] Le Blay P, Mouterde G, Barnetche T, Morel J, Combe B (2012). Short-term risk of total malignancy and nonmelanoma skin cancers with certolizumab and golimumab in patients with rheumatoid arthritis: metaanalysis of randomized controlled trials. J Rheumatol.

[24] Le Blay P, Mouterde G, Barnetche T, Morel J, Combe B (2012). Risk of malignancy including non-melanoma skin cancers with anti-tumor necrosis factor therapy in patients with rheumatoid arthritis: meta-analysis of registries and systematic review of long-term extension studies. Clin Exp Rheumatol.

[25] Askling J, Baecklund E, Granath F, Geborek P, Fored M, Backlin C (2009). Anti-tumour necrosis factor therapy in rheumatoid arthritis and risk of malignant lymphomas: relative risks and time trends in the Swedish biologics register. Ann Rheum Dis.

[26] Askling J, van Vollenhoven RF, Granath F, Raaschou P, Fored CM, Baecklund E (2009). Cancer risk in patients with rheumatoid arthritis treated with anti-tumor necrosis factor alpha therapies: does the risk change with the time since start of treatment?. Arthritis Rheum.

[27] Carmona L, Abasolo L, Descalzo MA, Perez-Zafrilla B, Sellas A, de Abajo F (2011). Cancer in patients with rheumatic diseases exposed to TNF antagonists. Sem Arthritis Rheum.

[28] Dixon WG, Watson KD, Lunt M, Mercer LK, Hyrich KL, Symmons DP (2010). Influence of anti-tumor necrosis factor therapy on cancer incidence in patients with rheumatoid arthritis who have had a prior malignancy: results from the British Society for Rheumatology biologics register. Arthritis Care Res.

[29] Dreyer L, Mellemkjaer L, Andersen AR, Bennett P, Poulsen UE, Juulsgaard Ellingsen T (2013). Incidences of overall and site specific cancers in TNFalpha inhibitor treated patients with rheumatoid arthritis and other arthritides - a follow-up study from the DANBIO registry. Ann Rheum Dis.

[30] Geborek P, Bladstrom A, Turesson C, Gulfe A, Petersson IF, Saxne T (2005). Tumour necrosis factor blockers do not increase overall tumour risk in patients with rheumatoid arthritis, but may be associated with an increased risk of lymphomas. Ann Rheum Dis.

[31] Pallavicini FB, Caporali R, Sarzi-Puttini P, Atzeni F, Bazzani C, Gorla R (2010). Tumour necrosis factor antagonist therapy and cancer development: analysis of the LORHEN registry. Autoimmunity Rev.

[32] Damjanov N, Nurmohamed MT, Szekanecz Z (2014). Biologics, cardiovascular effects and cancer. BMC Med.

[33] Mercer K, Lunt M, Low M, Dixon W, Watson K, Symmons D (2015). Risk of solid cancer in patients exposed to anti-tumour necrosis factor therapy: results from the British Society for Rheumatology biologics register for rheumatoid arthritis. Ann Rheum Dis.

[34] Wolfe F, Michaud K (2007). Biologic treatment of rheumatoid arthritis and the risk of malignancy: analyses from a large US observational study. Arthritis Rheum.

[35] Australian Bureau of Statistics. National Health Survey: First Results, 2014–15. Cat No 4364.0.55.001 Canberra Australia 2015 [Available from: http://www.abs.gov.au/ausstats/abs@.nsf/Lookup/by%20Subject/4364.0.55.001~2014-15~Main%20Features~Smoking~24.

[36] Raaschou P, Simard JF, Holmqvist M, Askling J (2013). Rheumatoid arthritis, anti-tumour necrosis factor therapy, and risk of malignant melanoma: nationwide population based prospective cohort study from Sweden. BMJ.

[37] Perkins S, Cohen M, Rahme E, Bernatsky S (2012). Melanoma and rheumatoid arthritis (brief report). Clin Rheumatol.

[38] Mercer LK, Askling J, Raaschou P, Dixon WG, Dreyer L, Hetland ML (2017). Risk of invasive melanoma in patients with rheumatoid arthritis treated with biologics: results from a collaborative project of 11 European biologic registers. Ann Rheum Dis.

[39] Wilton KM, Matteson EL (2017). Malignancy incidence, management, and prevention in patients with rheumatoid arthritis. Rheumatol Ther.

